# Anders Fredrik Regnell – an early and generous sponsor of our society

**DOI:** 10.3109/03009734.2011.555369

**Published:** 2011-02-11

**Authors:** Torbjörn Karlsson


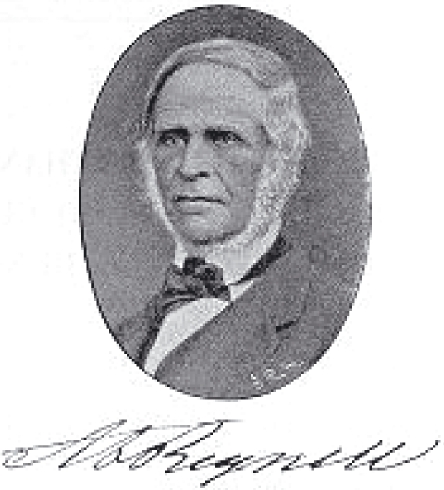


Borne in 1807 as the son of Anders Rignell, a horse driver and Brita Persdotter, a servant girl, Anders Fredrik became motherless at an early age. His father then married a rather wealthy widow and innkeeper, and with his new wife he was busy taking care of business. There wasn´t much time for family life for the young boy, but, he had the fortune of having a good education. Curiously, Anders Fredrik got his last name, Regnell, by a misspelling when enrolled in school. Anders Fredrik Regnell became a student in Uppsala 1824, passed the preparatory exams for medical studies 1830, med lic 1836 and got his medical doctorate as primus 1837. From 1836 to 1839 he worked in Stockholm and for some while as an assistant to Anders Retzius. He was appointed position as a Cholera physician twice.

1839-1840 he worked as a ship's doctor travelling with the Jarrama expedition to the Mediterranean regions. Returning home from this he was understandably concerned by his own health, with a lung disease causing blood coughing. Thus, he decided to emigrate to a warmer and healthier climate. In Rio de Janeiro he passed the tests of the medical faculty and was approved as a doctor. He then established a successful practice in the small town Caldas in Minas Geiras. Although he was something of a lonesome wolf he gave hospitality to many visitors from his old country.

As other well-known *medici* he showed an interest in botany. Later in life he collected plants in tropical regions, sending them to different museums in the old world to be characterized by experts. In South America he also showed interest in animal life, geology and meteorology.

Many great men have been remembered for their knowledge, contributions to science by new discoveries or by their synthesis of evolutions in science. Anders Fredrik Regnell deserves to be remembered also because of his monetary contributions to research and education. Due to his earnings as a skilled doctor and to wise investments in different projects *e g* mining he made a large fortune. Donations of impressive magnitude were made to many different organisations. First of all to his *Alma Mater*, Uppsala University and in fact Regnell's donations were most significant as they, next to those of Gustav II Adolf, were the largest ever given to Uppsala University. He gave money to the Swedish Society of Medicine and to the Swedish Museum of Natural History in Stockholm as well.

He also contributed to our society in a very substantial way. Among other things we should be greatful to Anders Fredrik Regnell for giving us the possibility to publish this journal at that time under the name of Upsala läkareförenings förhandlingar. He was a honorary member of our society, the Swedish Society of Medicine and of the learned societies in Uppsala and Gothenburg.

By one of his compatriots he was characterized as resembling “a cactus; rich in nutrient fluids with magnificent beautiful flowers, but, with a bizarre growth and filled with thorns”.

Bo Lindberg, a former chief physician at our hospital, this spring will publish a biography on the life of Anders Fredrik Regnell. The title of the book is “Anders Fredrik Regnell – läkare, botanist, donator”. Our society has contributed economically to the publication of this book, hard copies of which will be purchased by the University library. It will also be available electronically since it will be published under open access premises. I shall read this book with great interest, as the life of Anders Fredrik Regnell deserves to be remembered.

